# Analogous comparison unravels heightened antiviral defense and boosted viral infection upon immunosuppression in bat organoids

**DOI:** 10.1038/s41392-022-01247-w

**Published:** 2022-12-19

**Authors:** Xiaojuan Liu, Cun Li, Zhixin Wan, Man Chun Chiu, Jingjing Huang, Yifei Yu, Lin Zhu, Jian-Piao Cai, Lei Rong, You-qiang Song, Hin Chu, Zongwei Cai, Shibo Jiang, Kwok-yung Yuen, Jie Zhou

**Affiliations:** 1 Department of Microbiology, School of Clinical Medicine, Li Ka Shing Faculty of Medicine, The University of Hong Kong, Hong Kong, China; 2 State Key Laboratory of Environmental and Biological Analysis, Department of Chemistry, Hong Kong Baptist University, Hong Kong, China; 3 Department of Medicine, The University of Hong Kong-Shenzhen Hospital, Shenzhen, Guangdong China; 4 School of Biomedical Sciences, State Key Laboratory of Brain and Cognitive Sciences, The University of Hongkong, Hong Kong, China; 5 State Key Laboratory of Emerging Infectious Diseases, Carol Yu Centre for Infection, The University of Hong Kong, Hong Kong, China; 6 Centre for Virology, Vaccinology and Therapeutics, Hong Kong Science and Technology Park, Hong Kong, China; 7 Key Laboratory of Medical Molecular Virology (MOE/NHC/CAMS), Institute of Infectious Disease and Biosecurity, School of Basic Medical Sciences, Fudan University, Shanghai, China

**Keywords:** Microbiology, Intestinal stem cells

## Abstract

Horseshoe bats host numerous SARS-related coronaviruses without overt disease signs. Bat intestinal organoids, a unique model of bat intestinal epithelium, allow direct comparison with human intestinal organoids. We sought to unravel the cellular mechanism(s) underlying bat tolerance of coronaviruses by comparing the innate immunity in bat and human organoids. We optimized the culture medium, which enabled a consecutive passage of bat intestinal organoids for over one year. Basal expression levels of IFNs and IFN-stimulated genes were higher in bat organoids than in their human counterparts. Notably, bat organoids mounted a more rapid, robust and prolonged antiviral defense than human organoids upon Poly(I:C) stimulation. TLR3 and RLR might be the conserved pathways mediating antiviral response in bat and human intestinal organoids. The susceptibility of bat organoids to a bat coronavirus CoV-HKU4, but resistance to EV-71, an enterovirus of exclusive human origin, indicated that bat organoids adequately recapitulated the authentic susceptibility of bats to certain viruses. Importantly, TLR3/RLR inhibition in bat organoids significantly boosted viral growth in the early phase after SARS-CoV-2 or CoV-HKU4 infection. Collectively, the higher basal expression of antiviral genes, especially more rapid and robust induction of innate immune response, empowered bat cells to curtail virus propagation in the early phase of infection.

## Introduction

Bats are the natural reservoirs of diverse viruses associated with human diseases, including coronaviruses, filoviruses, lyssaviruses, and henipaviruses. In the past two decades, several outbreaks of coronavirus diseases have occurred, including SARS, MERS, and the current pandemic of COVID-19,^1^ all of which are invariably related to the spillover of bat-borne coronaviruses. The discovery of SARS-related coronaviruses in bats ignited an enthusiastic hunt for viruses in bats.^2^ As a result, bats have been identified as the richest source of diverse coronaviruses, including a school of bat coronaviruses, some of which were closely related to the viruses infective to humans or other animals.^3–5^ However, apart from lyssaviruses, bats appear to host viruses or coexist with viruses in an asymptomatic or paucisymptomatic manner.^6^ The fact that bats can asymptomatically host a diverse assortment of coronaviruses aroused an intriguing issue of why coronaviruses lead to distinct manifestations in humans and bats. A common feature in most human RNA virus infections is immunity-driven pathogenesis; aberrant immune responses elicited by viral infections cause tissue damage and disease symptoms.^7^ Generally, two non-exclusive viewpoints have been proposed to explain bat tolerance of viruses: bats may establish effective tolerance to virus propagation, or they maintain fitness through successful control of virus replication.

Prior knowledge of bat immunity is generally gained via three main approaches, i.e., comparative genomics and transcriptomic analyses, in vitro studies in bat cell lines, and experimental infections in bats. Although the essential components of innate and adaptive immune systems are conserved in bats,^8^ comparative genomics studies uncovered significant differences in bats. PYHIN family genes within the inflammasome pathway for recognizing DNA viruses and damaged self DNA, killer cell immunoglobulin-like receptors (KIR), and killer cell lectin-like receptors (KLR) are lost or significantly reduced in some bat species.^9,10^ In addition, contraction or expansion of IFN genes, expansion of APOBEC3 genes, positive selection of specific genes, and differences in essential protein domains were documented.^10,11^ Extensive interrogations of bat genomes have provided genomic evidence and hypotheses for further investigations. Nonetheless, gene expression profiles, especially gene expression kinetics upon viral infections, may dictate the disease manifestation and outcome. Indeed, bat immune defense against viruses and cellular interaction with viruses were analyzed in bat cell lines and bat immune cells.^12–14^ These studies converged on a general recognition of a dampened inflammatory response in bat cells.^13,14^ On the other hand, bat cell lines constitutively expressed IFNs and IFN-stimulated genes (ISGs),^15,16^ the basal transcriptional level of which was higher than that in human and mouse cell lines.

Bat-derived cell lines have provided insights into bat cellular biology in response to virus infections. However, similar to human cell lines, these bat cell lines might not be a good model for studying virus-host interactions since cell lines cultivated in vitro hardly model native cells in vivo. The third approach, experimental infections in bats, involves substantial challenges, especially in studying those viruses lethal to humans. Several captive bat colonies have been established for research purposes,^17^ yet are not readily accessible to most laboratories. Thus, a biologically relevant in vitro model of bat cells is urgently required. Animal coronaviruses commonly manifest as enteric infections.^18^ SARS-related coronaviruses were only detected in anal swabs of horseshoe bats,^2^ suggesting the enteric tropism of these viruses. Intestinal epithelial cells are the entry portal and primary infection site of enteric microbes. However, a robust protocol was barely available to cultivate and expand primary epithelial cells in vitro until the advent of organoid technology. Human intestinal organoids, the first adult stem cell-derived organoids, faithfully simulate the morphological and functional attributes of the human intestinal epithelium.^19^ These long-term expandable and physiologically-active human intestinal organoids have become a popular tool for studying infection of enteric microbes.^20–23^ Similar to coronavirus infections in animals, gastrointestinal involvement was reported in human coronavirus infections,^24,25^ although respiratory symptoms might be more notable. We and others have utilized human intestinal organoids to study MERS-CoV and SARS-CoV-2 enteric infection.^23–27^ In addition, we established the first bat intestinal organoids from the Chinese horseshoe bats and demonstrated productive SARS-CoV-2 infection in these bat organoids.^24^

Coronaviruses exhibited enteric tropism in humans and animals; intestinal epithelial cells are situated at the frontline to encounter virus invasion. The susceptibility of these epithelial cells to particular viruses dictates the host and tissue tropism of the viruses. Meanwhile, epithelial cells elicit an innate immune response upon viral infections, which subsequently triggers a cascade of host responses to counteract virus invasion and maintain homeostasis.^22^ As such, the interaction between viruses and host intestinal epithelial cells contributes substantially to viral tropism, viral pathogenesis and disease manifestation in the hosts. However, the lack of a robust in vitro model has seriously hampered the dissection of virus-host interaction and virus-induced pathogenesis in intestinal epithelial cells. Here, with the unique bat intestinal organoid culture system established in our lab, we conducted a comparative study in bat and human intestinal organoids, aiming to reveal the cellular response in the organoids and shed light on the biological basis for bat asymptomatically hosting viruses.

## Results

### Optimizing and characterizing bat intestinal organoids

**Fig. 1:**
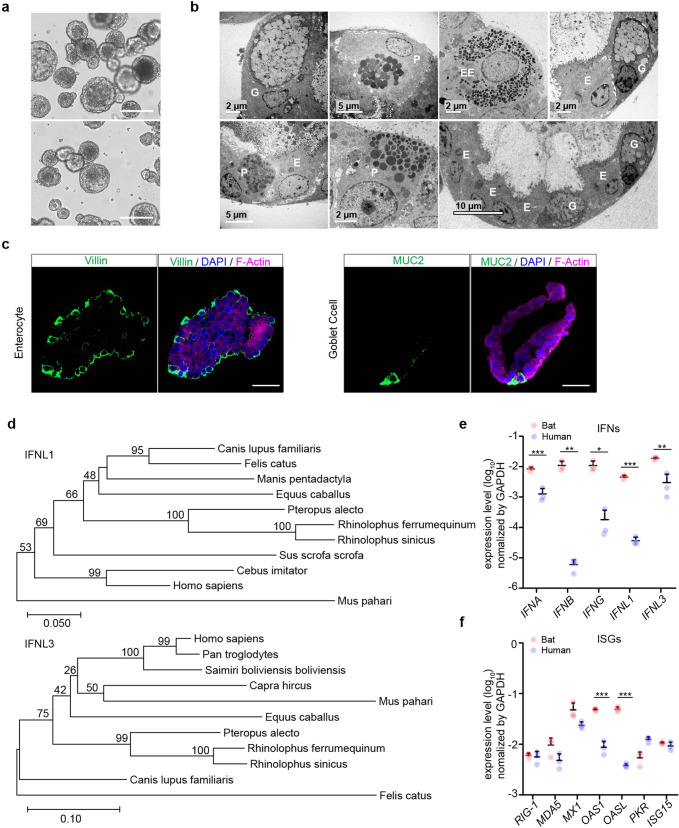
Characterization of optimized bat intestinal organoids and detection of the basal expression of antiviral genes. **a** Photomicrographs of optimized bat intestinal organoids on day 7 after passaging (magnification ×100). Scale bar, 100 μm. **b** Transmission electron microscopy illustrates the ultrastructural morphology of absorptive enterocyte (E), Paneath cell (P), goblet cell (G), and enteroendocrine cell (EE) in bat intestinal organoids. **c** Bat intestinal organoids were fixed and immunostained to label Villin + (green) enterocyte and MUC2 + (green) goblet cell. Nuclei and actin filaments were counterstained with DAPI (blue) and Phalloidin-647 (purple), respectively. Scale bar, 20 µm. **d** Phylogenetic analysis based on an alignment of horseshoe bat (*Rhinolophus sinicus*) *IFNL1* and *IFNL3* ORF cDNAs with other Chiroptera species and mammals. Bootstrap values (%) are indicated on each branch, and the scale for branch length is shown at the bottom of the tree. **e**, **f** GAPDH-normalized expression levels of IFN genes (**e**) and ISGs (**f**) in bat and human intestinal organoids. Data represent the mean and s.d. of a representative experiment in organoids from a bat and human donor, *n* = 3. Two-tailed unpaired Student’s *t* test

Intestinal organoids were derived from horseshoe bats with a perfect establishment rate as we reported previously^24^ and consecutively passaged for around 3 months when cultured in the medium supplemented with a Wnt3a conditioned medium. We optimized the composition of the culture medium to lengthen the expandability of bat intestinal organoids and have a greater amount of stable bat organoids for experimentation and reduce the usage of live bats. A next-generation surrogate Wnt agonist was reported to support the growth of human intestinal organoids more efficiently than the Wnt3a conditioned medium.^28^ Thus, we switched to a modified culture medium (Supplementary Table 1) in which the Wnt surrogate replaced the original Wnt3a conditioned medium. The addition of the Wnt surrogate sustained a dramatically prolonged expansion of bat intestinal organoids for over one year. We routinely maintained 3 lines of bat organoids derived from 3 horseshoe bats (*Rhinolophus sinicus*) and passaged these organoids every 7 days for daily experimentation (Fig. 1a). Transmission electron microscopy revealed that the bat intestinal organoids consisted of enterocyte, goblet cell, Paneth cell and enteroendocrine cell (Fig. 1b); goblet cell, especially enterocyte, were the dominant cell populations in bat intestinal organoids (the bottom right panel in Fig. 1b), consistent with the many prior studies in human intestinal organoids.^19,24,26^ Due to the lack of specific antibodies against bat proteins, we used cell-type specific antibodies against human analogs. Immunostaining using human cell-type specific antibodies verified the presence of MUC2 + goblet cells and abundant Villin+ enterocytes in bat intestinal organoids (Fig. 1c). Thus, the optimized bat intestinal organoids are long-term expandable and faithfully simulate the bat intestinal epithelium. The comparable cellular composition of bat and human intestinal organoids provided a unique and biologically-active model system, enabling an analogous comparison of bat and human intestinal epithelial cells in vitro.

### Detecting the basal expression of antiviral genes in bat intestinal organoids

Type III interferons (IFN) are the major player of mucosal immunity in the intestines of human and mouse.^29^ IFN lambda-1 (IFNL1) and IFNL3 were highly induced in human intestinal organoids upon viral infections.^21,26^ We first determined the sequences of horseshoe bat type III IFNs using RACE PCR. A long coverage genome sequence of the Chinese horseshoe bat (*Rhinolophus sinicus)* is publicly available in the GenBank Bioproject database (Accession No. PRJNA294852). Sequences of analogous human genes, including IFNL1 and IFNL3, were blast searched in the genome of Chinese horseshoe bat. The matched sequences were used to design RACE PCR primers (Supplementary Table 2) to detect partial sequences. Then 5’ and 3’ RACE PCR assay was performed using RNA extracted from bat intestinal organoids. We obtained two sequences with high homology to interferon lambda-1 like and interferon lambda-3 like genes in the closely related bat species *Rhinolophus ferrumenguinum*, which were designated IFNL1 and IFNL3, respectively. A comparison of the identified IFNLs with other mammals demonstrated that IFNL1 and IFNL3 of *Rhinolophus sinicus* were identical to those of *Rhinolophus ferrumequinum* and 99% homologous to those of *Pteropus alecto* (Fig. 1d).

We also designed qPCR primers using the same approach and examined genes of innate immunity in bat intestinal organoids. We examined the basal levels of IFNs and several reported ISGs^15^ in bat intestinal organoids and compared them with those in the human organoids that were cultured with the same protocol (Fig. 1e). We found that bat organoids expressed all three types of IFNs with 1–3 log units higher than human organoids. Several ISGs such as OAS1 and OASL were approximately 10-fold higher in bat organoids than their human counterparts, while others, such as MX1, and MDA5 that were IFN-inducible in bat cells,^16^ tended to be expressed higher in bat organoids (Fig. 1f). Overall, basal expression levels of IFNs and some ISGs were significantly higher in bat intestinal organoids than in their human counterparts.

### Bat organoids eliciting a more robust antiviral response to Poly(I:C) treatment compared to human organoids

**Fig. 2:**
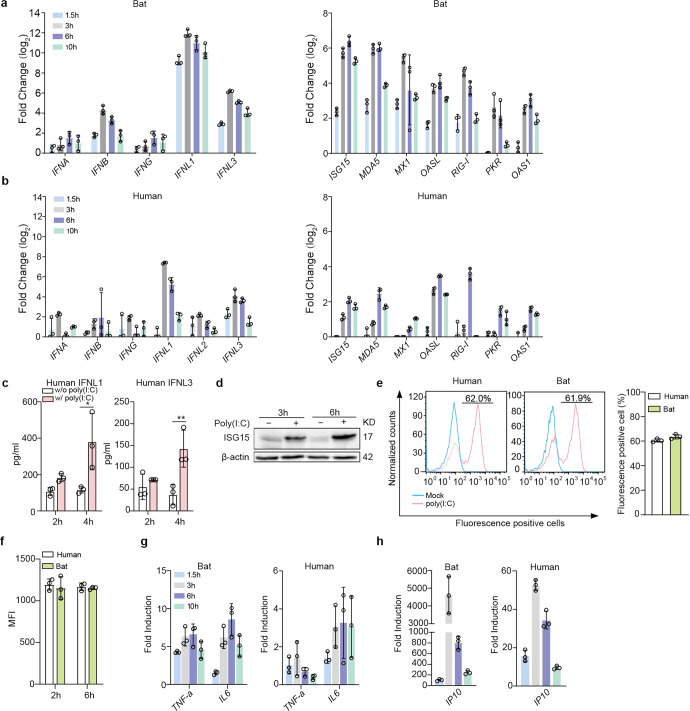
Bat organoids elicited a more robust antiviral response to Poly(I:C) treatment. Bat and human intestinal organoids were sheared mechanically and treated with 10 μg/ml Poly(I:C) or mock-treated with DMSO. **a**, **b** Induction of IFNs (left) and ISGs (right) in bat intestinal organoids (**a**) and human intestinal organoids (**b**) at the indicated hours after Poly(I:C) treatment. Results show the log2-fold change of GAPDH-normalized expression level in the treated organoids relative to mock-treated organoids. Data represent the mean and s.d. of a representative experiment in organoids from a bat and human donor, *n* = 3. **c** Culture media from the treated or mock-treated human intestinal organoids at the indicated hours were applied to ELISA to measure concentrations of IFNL1 and IFNL3. Data represent the mean and s.d. of a representative experiment in one line of human organoids, n = 3. Two-tailed unpaired Student’s *t* test. **d** Poly(I:C) treated or mock-treated human intestinal organoids were collected at the indicated hours post-treatment and subjected to Western blot to detect human ISG15. **e**, **f** Bat and human intestinal organoids were sheared and incubated with 10 μg/ml Poly(I:C) Fluorescein in triplicate for 2 h and 6 h. The organoids were then dissociated and applied to flow cytometry to detect the percentage at 2 h post treatment (**e**) and mean fluorescence intensity (MFI, **f**) of Fluorescein-positive cells at 2 and 6 h post treatment. **g**, **h** Induction of TNF-a and IL6 (**g**) and IP10 (**h**) in bat and human intestinal organoids at the indicated hours after treatment. Results show the fold change of GAPDH-normalized expression level in Poly(I:C)-treated organoids relative to mock-treated organoids. Data represent the mean and s.d. of a representative experiment in organoids from a bat and human donor, *n* = 3

We then analyzed the induction of antiviral response in bat and human intestinal organoids. Poly(I:C), a synthetic virus mimic, induced innate immunity in human intestinal cells and intestinal organoids through binding to TLR3 and MDA5,^30^ pattern recognition receptors on human intestinal cells.^31^ First, we tested the conditions to deliver Poly(I:C) to bat intestinal organoids for a robust induction of cellular response. We found that incubation of bat organoids with 10 µg/ml Poly(I:C) after mechanical shearing consistently and effectively induced antiviral genes (Supplementary Fig. 1). We then incubated bat and human intestinal organoids in parallel with or without 10 µg/ml Poly(I:C) after shearing and examined expression levels of antiviral genes in bat and human intestinal organoids relative to those in mock-treated organoids. Type III IFNs, especially IFNL1, were highly induced in bat organoids after Poly(I:C) stimulation, whereas type I and II IFNs, including IFN-α (IFNA), -β (IFNB) and -γ (IFNG), were modestly induced, with a much lower magnitude than that of IFNL1 and IFNL3 (Fig. 2a), very consistent with the previous findings of type III IFNs as a major player in mucosal immunity of human and mouse.^21,26^ Several ISGs, including ISG15, MX1 and MDA5, were highly upregulated with a slight delay compared to IFNs (Fig. 2a). Human intestinal organoids displayed a similar profile of immune activation. However, the induction of type III IFNs and ISGs, was more rapid and potent, and sustained longer in bat organoids than in their human counterparts (Fig. 2b). More active and prolonged induction of antiviral defense in bat organoids was observed in multiple experiments comparing bat and human intestinal organoids from different donors (Supplementary Fig. 2). The secretion of human IFNL1 and IFNL3 from Poly(I:C)-treated human intestinal organoids was verified by ELISA (Fig. 2c), and Poly(I:C)-induced upregulation of human ISG15 was shown by Western blotting (Fig. 2d and Supplementary Fig. 6). The higher magnitude of innate immune activation in bat organoids than in human organoids was intrinsic since bat and human organoids showed a comparable uptake of fluorescein-labeled Poly(I:C) as determined by flow cytometry analysis. The proportion of fluorescein-positive cells and the fluorescence intensity were comparable in bat and human intestinal organoids after incubation with fluorescein-labeled Poly(I:C) (Fig. 2e, f and Supplementary Figs. 3 and 6).

Poly(I:C) is a dual agonist of TLR3 and MDA5.^30^ Apart from IFN induction, TLR3 or MDA5 activation also leads to the production of proinflammatory cytokines through NF-κB signaling. We examined proinflammatory cytokines and found that IL6 and TNF-α were induced in bat intestinal organoids (left panel, Fig. 2g). Notably, IP10 was rapidly and dramatically induced after Poly(I:C) treatment (left panel, Fig. 2h). Human intestinal organoids showed similar induction kinetics, yet with a much lower magnitude than bat organoids (right panels, Fig. 2g, h).

### TLR3 and RLR signaling pathways mediating antiviral response in bat and human organoids

**Fig. 3:**
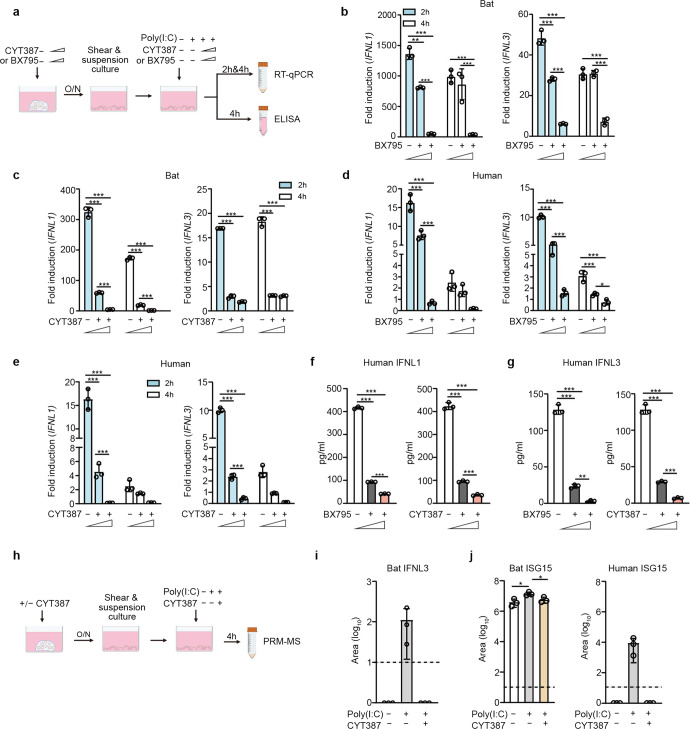
TLR3 and RLR signaling pathways mediate antiviral responses in bat and human organoids. **a** A schematic graph outlines the experimental procedure for panels **b**–**g**. Bat and human intestinal organoids were pretreated with BX795 (0, 0.1 and 1 μM) or CYT387 (0, 0.1, and 1 μg/ml) overnight. After mechanical shearing, the organoids were incubated with or without 10 μg/ml Poly(I:C), together with the initial concentrations of BX795 or CYT387. At the indicated hours after Poly(I:C) treatment, the organoids were harvested and subjected to RT-qPCR assay to examine mRNA expression levels of bat and human IFNL1 and IFNL3; cell-free media from human organoids were applied to ELISA to detect IFNL1 and IFNL3. Data represent the mean and s.d. of a representative experiment in organoids from a bat and human donor, *n* = 3. Ordinary one-way ANOVA with Tukey’s multiple comparison test. **b**, **c** Induction of IFNL1 (left) and IFNL3 (right) in bat intestinal organoids treated with BX795 (**b**) and CYT387 (**c**). Results show the fold change of GAPDH-normalized expression level in the Poly(I:C)-treated organoids relative to mock-treated organoids. **d**, **e** Induction of IFNL1 and IFNL3 in human intestinal organoids treated with BX795 (**d**) and CYT387 (**e**). IFNL1 (**f**) and IFNL3 (**g**) secretion from the human organoids at 4 h post-stimulation. **h** A schematic graph describes the experimental procedure for panels **i** and **j**. Bat and human intestinal organoids were pretreated with 1 μg/ml CYT387 or DMSO overnight. After mechanical shearing, the organoids were incubated with 1 μg/ml CYT387 or DMSO with or without 10 μg/ml Poly(I:C) for 4 h. Cell-free media were then harvested and subjected to PRM-MS to analyze bat IFNL3 (**i**) and ISG15 of bat and human (**j**). The dotted lines represent the detection limit. Data represent the mean and s.d. of a representative experiment in organoids from a bat and human donor, *n* = 3. Two-tailed unpaired Student’s *t* test

We proceeded to elucidate signaling pathway(s) mediating immune activation in bat organoids. As aforementioned, Poly(I:C) is a synthetic agonist of TLR3 and MDA5. BX795 is a catalytic inhibitor of TBK1/IKKε, a kinase in TLR3 and RLR pathways. CYT387, a potent inhibitor of TBK1/IKKε, can also inhibit JAK1 and JAK2,^32^ Janus kinases in the JAK/STAT pathway mediating IFN signaling. We first determined the appropriate concentrations of the two inhibitors to exclude the potential artifact of cytotoxicity confounding the readouts in bat and human organoids (Supplementary Fig. 4). Bat and human intestinal organoids were pretreated with BX795 or CYT387 or DMSO overnight with concentrations of minimal cytotoxicity, followed by Poly(I:C) stimulation or mock stimulation and further incubation with the initial concentrations of BX795 or CYT387 or DMSO (Fig. 3a). We then examined IFNL1 and IFNL3 induction in the organoids at 2 and 4 h after Poly(I:C) stimulation relative to those with mock stimulation. BX795 inhibition suppressed Poly(I:C) induced bat IFNL1 and IFNL3 in a dose-dependent manner (Fig. 3b). CYT387 appeared to have a more potent inhibitory effect on Poly(I:C)-triggered induction of IFNL1 and IFNL3 in bat organoids (Fig. 3c). Similarly, BX795 (Fig. 3d) and CYT387 (Fig. 3e) treatment abrogated Poly(I:C)-stimulated IFNL1 and IFNL3 upregulation in human intestinal organoids. Again, Poly(I:C)-stimulated IFNL1 and IFNL3 production less intensively in human organoids than in bat intestinal organoids. In addition, Poly(I:C)-triggered IFNL1 (Fig. 3f) and IFNL3 (Fig. 3g) secretion from human organoids decreased in a dose-dependent manner with increasing concentrations of BX795 and CYT387.

We developed a parallel reaction monitoring mass spectrometry (PRM-MS) assay for targeted quantification of bat IFNL3 protein and ISG15 protein in the culture medium of bat and human organoids in the presence or absence of CYT387 and Poly(I:C) treatment (Fig. 3h), due to the lack of specific antibodies for detecting bat proteins. The result verified Poly(I:C)-triggered secretion of IFNL3 in bat organoids, which was nullified by CYT387 treatment (Fig. 3i). However, we failed to detect bat IFNL1 by PRM-MS, although the qPCR assay showed a higher induction than IFNL3. The failed identification, we inferred, might be related to the technical issue, rather than the absence of IFNL1 protein in bat organoids. We also measured ISG15 secretion from the bat and human organoids by PRM-MS. Bat ISG15 secreted from Poly(I:C)-treated bat organoids was significantly higher than that in mock-treated organoids, and significantly lower than that in Poly(I:C) + CYT387 double-treated organoids (Fig. 3j, left panel). ISG15 secretion from human organoids was only detectable in Poly(I:C) treated organoids, that in mock-treated organoids and Poly(I:C) + CYT387 double-treated organoids was below the detection limit (Fig. 3j, right panel). Thus, CYT387 treatment substantially dampened bat and human ISG15 production stimulated by Poly(I:C) (Fig. 3j). Here, we had to select specific and distinct peptides to quantify human and bat ISG15, which may result in different detection limits and dynamic ranges for the targeted peptides. Thus, it would be biased to directly compare the abundance of bat and human ISG15, and the magnitude of their induction. Overall, BX795 and CYT387 nullified Poly(I:C)-triggered induction of IFNLs and ISG in both bat and human intestinal organoids, suggesting that TLR3 and RLR signaling might be the conserved pathways mediating antiviral response in bat and human intestinal epithelial cells.

### Bat intestinal organoids recapitulating bat susceptibility to viruses

**Fig. 4:**
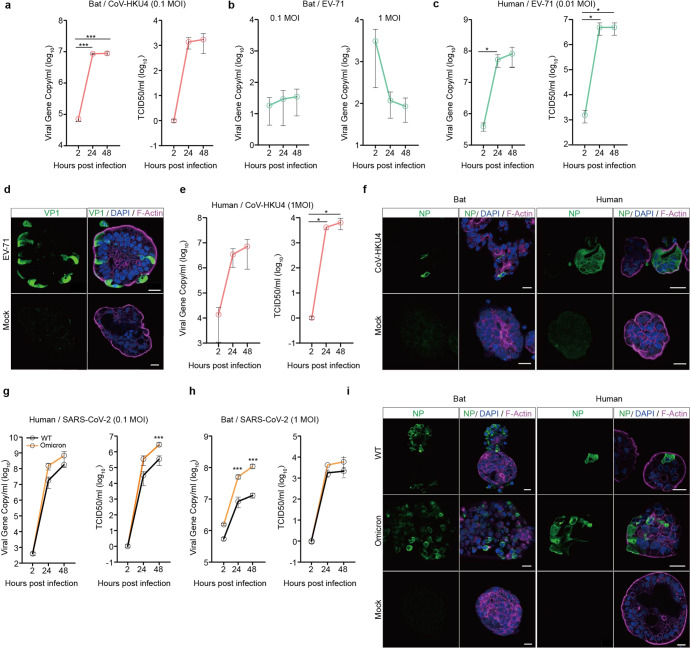
Bat intestinal organoids reproduced bat susceptibility to coronaviruses. **a**–**c** At the indicated hours after bat intestinal organoids inoculated with CoV-HKU4 (**a**), EV-71 (**b**) and human intestinal organoids inoculated with EV-71 (**c**), culture media were harvested and applied to viral load detection by RT-qPCR and viral titration by TCID50 assay. Data represent mean and s.d. in a representative experiment, *n* = 3. Two-tailed unpaired Student’s *t* test. **d** EV-71 infected and mock-infected human intestinal organoids were fixed and immunostained to identify viral protein VP1 positive (green) cells. Nuclei and actin filaments were counterstained with DAPI (blue) and Phalloidin-647 (purple), respectively. Scale bar, 20 µm. **e** At the indicated hours after human intestinal organoids inoculated with CoV-HKU4, culture media were harvested and applied to viral load detection and viral titration. Data represent mean and s.d. in a representative experiment, *n* = 3. Two-tailed unpaired Student’s *t* test. **f** Bat and human intestinal organoids infected with CoV-HKU4 or mock-infected were fixed and immunostained to identify CoV-HKU4 NP positive (green) cells. Scale bar, 20 µm. **g**, **h** At the indicated hours after human and bat intestinal organoids inoculated with WT SARS-CoV-2 and the Omicron variant, culture media were harvested and applied to viral load detection and viral titration. Data represent mean and s.d. in a representative experiment, *n* = 3. Ordinary one-way ANOVA with Tukey’s multiple comparison test. **i** SARS-CoV-2 infected and mock-infected bat and human intestinal organoids were fixed and immunolabeled to identify SARS-CoV-2 NP positive (green) cells. Scale bar, 20 µm

Given that bat intestinal organoids simulated bat intestinal epithelium, we inferred that bat intestinal organoids might be able to reproduce the authentic susceptibility of bat intestinal cells to coronaviruses. Despite the discovery of many bat coronaviruses, only a couple of them have been successfully isolated and cultivated or propagated from molecularly engineered viruses.^5,33^ A lineage C beta-coronavirus *Tylonycteris pachypus* coronavirus HKU4 (CoV-HKU4) is one of the cultivable bat coronaviruses, probably owing to its usage of human DPP4 as its cellular receptor.^34^ In contrast, enterovirus 71 (EV-71) has a limited host range; humans are the only known natural host. We inferred that bat intestinal organoids might be susceptible to CoV-HKU4, but non-susceptible to EV-71. To this end, we tested CoV-HKU4 and EV-71 in bat intestinal organoids. As shown in Fig. 4a, CoV-HKU4 displayed a significantly increased viral gene copy and viral titer after inoculation. Within 48 h post-inoculation, infectious virions in the culture media increased by more than 3 log units. In contrast, we did not observe any sign of viral propagation, no matter whether the bat organoids were exposed to a high or low dose of EV-71 inoculum (Fig. 4b). After a high MOI inoculation (MOI of 1), viral load even decreased gradually after infection, suggesting that bat organoids were non-permissive to the enteric virus of human origin. EV-71 was inoculated in human intestinal organoids as a positive control and displayed a robust viral propagation (Fig. 4c, d), congruent with previous reports.^21,26^ Notably, CoV-HKU4 also replicated in human intestinal organoids (Fig. 4e), echoing the previous finding of its productive infection in human DPP4 transgenic mice.^35^ CoV-HKU4 infection in bat and human intestinal organoids was verified by immunofluorescence staining (Fig. 4f) using an antiserum against CoV-HKU4 nucleocapsid protein (NP).

RaTG13, a bat coronavirus with 96% homology to SARS-CoV-2, was identified in the droppings of horseshoe bats *Rhinolophus affinis*.^1^ Similar to most bat viruses, RaTG13 was not isolated and cultivated. Without live RaTG13 virus, we tested SARS-CoV-2 and found that intestinal organoids derived from horseshoe bats were permissive to SARS-CoV-2 viruses, both the ancestral strain (WT) and the more infectious Omicron variant that emerged in late 2021. Nevertheless, SARS-CoV-2 viruses replicated more actively in human intestinal organoids (Fig. 4g) than in bat organoids (Fig. 4h). Figure 4h showed the replication kinetics in bat organoids after an MOI of 1 inoculation. After inoculation with an MOI of 0.1, the same MOI for inoculating human organoids, the viruses barely replicated in bat organoids (data not shown). Interestingly, the more infectious Omicron variant appeared to possess a higher replicative fitness than the ancestral WT virus in both bat and human organoids. More productive infections of the WT strain and the Omicron variant in human than bat intestinal organoids were verified by immunofluorescence staining (Fig. 4i). Overall, the distinct permissiveness of bat intestinal organoids to a bat coronavirus and an enteric virus of exclusive human origin indicated that bat intestinal organoids recapitulated the authentic susceptibility of live bats to particular viruses. SARS-CoV-2 replication in bat intestinal organoids further supported that bat organoids served as an in vitro correlate of bat intestinal epithelium for phenocopying viral tropism in bat intestines.

We also measured the virus-induced innate immune response in human and bat intestinal organoids. At 48 h post inoculation of SARS-CoV-2, we harvested the human and bat organoids to detect the transactivation of antiviral genes and proinflammatory cytokines. Type III IFNs were highly induced in SARS-CoV-2-infected human organoids (Supplementary Fig. 5), which was consistent to our previous observations^24,26^ as well as their activation after Poly(I:C) treatment (Fig. 2b). ISGs, TNF-α and IP10 showed a similar induction profile as that in Poly(I:C)-treated human organoids (Supplementary Fig. 5). However, we did not observe a notable induction of these innate immune genes in bat organoids after SARS-CoV-2 infection (data not shown). It, we believe, might be related to the less productive viral replication in bat organoids (Fig. 4g, h).

### CYT387 treatment boosting early viral propagation in bat organoids

**Fig. 5:**
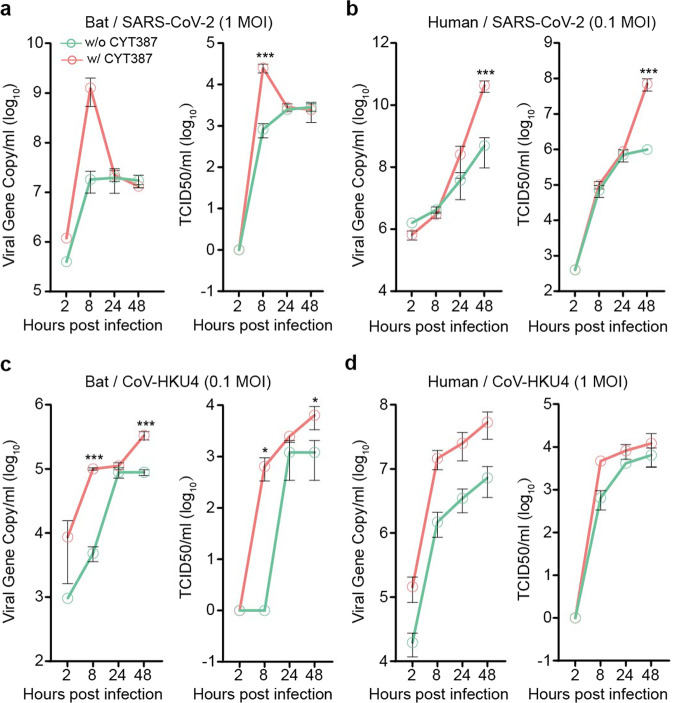
CYT387 treatment enhanced viral replication in bat intestinal organoids in the early stage of viral growth. Bat and human intestinal organoids were pretreated with 1 μg/ml CYT387 or DMSO overnight. After inoculation with WT SARS-CoV-2 or CoV-HKU4, the pretreated bat and human organoids were incubated with 1 μg/ml CYT387 or DMSO. At indicated time points after infection, cell-free media were collected and subjected to viral load detection and viral titration. Data represent mean and s.d. in each organoid line, *n* = 3. Ordinary one-way ANOVA with Tukey’s multiple comparison test. Viral load and viral titer of SARS-CoV-2 in treated and mock-treated bat organoids (**a**) and human organoids (**b**). Viral load and viral titer of CoV-HKU4 in treated and mock-treated bat organoids (**c**) and human organoids (**d**)

Now that bat intestinal organoids possessed a higher basal level of antiviral defense than their human counterparts and mounted a more active immune induction upon Poly(I:C) treatment, perturbation of host antiviral defense in bat and human organoids might affect viral growth disparately. Based on the more potent immunosuppressive effect of CYT387 than BX795 as revealed in Fig. 3, we treated bat and human organoids with CYT387, and assessed the impact of blunted immune activation during virus infections. After pretreatment with CYT387 or DMSO overnight, we inoculated bat and human organoids with SARS-CoV-2 and then incubated the organoids with the initial concentrations of CYT387 and DMSO accordingly. We monitored viral growth by detecting the viral load and viral titer in the culture media. As shown in Fig. 5a, CYT387 substantially enhanced viral growth at 8 h post-infection in bat organoids, whereas CYT387 enhancement of viral growth in human organoids occurred at 48 h (Fig. 5b). We performed similar experiments with CoV-HKU4 since both human and bat intestinal organoids sustained CoV-HKU4 replication. The result reproduced a significant boost of viral growth empowered by CYT387 in bat organoids at the early phase of infection (Fig. 5c). However, CYT387-mediated viral enhancement of CoV-HKU4 in human organoids was not as remarkable as that in bat organoids (Fig. 5d). Overall, the results indicated that bat organoids possessed a more robust antiviral machinery enabling instant control of virus replication in the early phase of infection.

## Discussion

Bats have been associated with infectious diseases for centuries. Growing evidence indicated that bats are the richest source of diverse coronaviruses, some of which are linked to significant diseases in humans and other mammals,^18^ including the ongoing COVID-19 pandemic.^1^ However, bats lacked disease signs even when experimentally infected with coronaviruses.^36^ Tremendous efforts have been made to explore the biological mechanisms that enable bats to host diverse viruses, especially coronaviruses that are pathogenic in humans, in a disease-free manner. Comparative genomic studies in bats uncovered loss of immunity-related genes, as well as expansion or contraction of antiviral genes.^9–11,37^ Studies in bat cell lines or bat primary immune cells demonstrated higher basal expression levels of IFNs and ISGs and dampened proinflammatory responses compared to those in human and mouse cells.^13,14^

Coronaviruses exhibit an enteric tropism in animals and humans, including SARS-related coronaviruses carried by horseshoe bats^2^ and human coronaviruses such as SARS-CoV, MERS-CoV, and SARS-CoV-2.^24,25,38^ Intestinal epithelial cells are the entry portal and primary infection site of enteric viruses. The ability of a virus to infect and replicate in these epithelial cells dictates its host and tissue tropism; meanwhile, the innate immune response elicited by infected epithelial cells orchestrates a cascade of host defense to counteract the invading viruses and maintain hemostasis. Immune cells are recruited subsequently in the arms race as a result of inflammatory signals released from infected epithelial cells. However, most knowledge of host response to microbial infections has been gathered from research on hematopoietic immune cells or the derived cells (e.g., monocyte-derived macrophages), mainly attributable to the relatively easier accessibility and mature methodologies to culture these cells. The bottleneck to understanding the host response in mucosal epithelial cells, the primary targets of invading viruses, is the lack of primary epithelial cells for routine experimentation. Under the conventional adhesion culture, primary epithelial cells have a limited proliferation capacity in vitro and eventually enter senescence, known as Hayflick limit.^39^ Many cell lines, including several bat cell lines, were derived from primary tissues such as the kidney or lung.^12^ Apart from epithelial cells, the source tissues themselves contain multiple cell types with variable replicative capacity under the routine adherent monolayer culture. In addition, during the procedure to derive cell lines, cells extracted from native tissues undergo an array of biological alterations in order to adapt and grow on the plastic surface of culture plates.^17,40,41^ Thus, the ultimate derivation of cell lines represents the survivorship of adapted and altered cells. The resultant cell lines are invariably homogeneous and very distinct from in vivo epithelial cells in terms of cellular identity and biological functionality. The advances in organoid technology have provided an excellent solution for this long-standing obstacle. The first adult stem cell-derived organoids, human intestinal organoids, faithfully simulate the morphological and functional attributes of the human intestinal epithelial cells,^19,42^ thus becoming a popular tool for studying virus-host interactions.^20^

We previously established bat intestinal organoids from Chinese horseshoe bats that abundantly host SARS-related coronaviruses.^2,24^ Bat intestinal organoids contained four major epithelial cell types in bat intestinal mucosa, and were consecutively propagated for around 3 months. In this study, we found that the modified culture medium dramatically increased the expandability of bat intestinal organoids and enabled a consecutive passage for over one year. We now routinely passage bat organoids every 7 days with a ratio of 1:3~5 (Fig. 1). The optimized bat organoids showed a comparable cellular composition to human intestinal organoids, providing a very stable source and generating a sufficient amount of bat intestinal epithelial cells for daily experimentation. Moreover, we have human intestinal organoids for comparative studies. These intestinal organoids represent a unique and biologically-active model system that enables an analogous comparison of bat and human intestinal epithelial cells, which would reveal potential differences in cellular immunity.

The comparison of bat and human organoids indicated that bat organoids did show a higher basal level of IFNs and some ISGs than human organoids (Fig. 1e, f), as reported previously in bat primary immune cells and bat cell lines.^15,16^ Of note, we found IFNs and ISGs were differentially induced in bat and human organoids in response to Poly(I:C) stimulation (Fig. 2). Bat organoids elicited a more robust antiviral response. Among all IFN molecules, IFNL3, especially IFNL1, were highly induced with a substantially higher magnitude than type I and II IFNs (Fig. 2a). The results indicate that type III IFNs are the major player in bat mucosal immunity, similar to the previous findings in human intestinal organoids and intestines of experimental mice.^21,26^ Notably, the antiviral defense spearheaded by type III IFNs in bat organoids outperformed their human counterparts in terms of swiftness and robustness (Fig. 2b). Poly(I:C) stimulation also triggered the induction of ISGs and proinflammatory cytokines and chemokines such as IP10, IL6, and TNF-α in bat organoids, with a higher magnitude than in human organoids (Fig. 2g, h). It was an unbiased comparison between bat and human organoids, given the comparable incorporation of Poly(I:C) into the organoids of two species (Fig. 2e, f and Supplementary Fig. 3). However, prior studies in bat cell lines suggested that a transcriptional repressor cRel suppressed Poly(I:C)-induced TNF-α activation, unlike the human cell line where TNF-α was highly induced.^43^ It was also documented that cRel underwent positive selection in other bat species,^9^ suggesting that it might be a common mechanism to attenuate inflammation. Here, our results revealed a distinct pattern of TNF-α induction in bat intestinal organoids from horseshoe bats. We will further investigate whether the activation of the proinflammatory cytokines is operational in the organoids from other bat species.

RaTG13, a bat coronavirus with 96% genome sequence identity to SARS-CoV-2, was identified in the droppings of horseshoe bats *Rhinolophus affinis*.^1^ Without live RaTG13 virus, we found that the intestinal organoids derived from horseshoe bats *Rhinolophus sinicus* were permissive to SARS-CoV-2 and sustained active virus growth, which might be attributed to SARS-CoV-2 utilization of ACE2 of horseshoe bats and the high homology of SARS-CoV-2 with RaTG13.^1^ We inferred that these bat intestinal organoids might reproduce the host and tissue tropism of SARS-related coronaviruses in authentic horseshoe bats. Here, we provided further evidence that bat intestinal organoids adequately modeled bat permissiveness to viruses. Bat intestinal organoids were susceptible to a bat beta-coronavirus CoV-HKU4, but resistant to EV-71, an enterovirus of exclusive human origin (Fig. 4). Thus, bat intestinal organoids could serve as an in vitro correlate of bat intestinal epithelium for phenocopying the host and tissue tropism of bat viruses, and provide direct wet-lab evidence on whether bats are the natural host of zoonotic viruses. However, studies in bat cell lines indicated that their permissiveness to the Ebola virus did not correlate with authentic host tropism. The tested cell lines derived from Egyptian *rousette* bats sustained an active replication of the Ebola virus, to which Egyptian *rousette* bats were actually refractory.^44^ The results alert us to be cautious about the biological relevance of bat cell lines; the immortalized bat cell lines may vary substantially from in vivo cells. Interestingly, we found that bat-borne beta-coronavirus CoV-HKU4 replicated in human intestinal organoids, consistent with its usage of human DPP4 as a receptor. Afterward, we would test CoV-HKU4 in our human respiratory organoids,^45–47^ the first adult stem cell-derived human respiratory organoids, to assess the zoonotic potential of CoV-HKU4.

After Poly(I:C) stimulation, bat intestinal organoids elicited a more rapid antiviral defense with a higher magnitude than the human counterparts. Using two synthetic inhibitors, we found that bat and human intestinal organoids may share similar TLR3 and RLR pathways to elicit antiviral immunity since the two inhibitors efficiently suppressed Poly(I:C)-triggered induction of type III IFNs and ISGs in bat and human organoids (Fig. 3). SARS-CoV-2 infection triggered a similar induction profile of antiviral and proinflammatory genes in human intestinal organoids (Supplementary Fig. 5) to that seen in Poly(I:C) stimulation. However, SARS-CoV-2-infected bat organoids did not show a notable induction of the innate immune genes as detected by RT-qPCR assay. We ascribed this to the less productive viral replication in bat organoids. The absolute readings of viral load/titer and the increment of viral load/titer over time were lower in bat organoids than in human organoids (Fig. 4g, h). In a less productive infection, most uninfected cells remained unstimulated; cellular response might be triggered in a small proportion of infected cells, yet masked in the RT-qPCR assay of a whole batch of organoids. This was actually the rationale for us to compare Poly(I:C)-triggered innate immunity in human and bat organoids, i.e., a comparable incorporation of the virus mimic into both organoids. Nevertheless, being unable to demonstrate virus-induced host response in bat organoids is indeed a limitation of the study. As such, we may have to perform single-cell sequencing later for an in-depth interrogation to detect the potential immune activation in bat organoids. Similarly, we could not present data to demonstrate TLR3/RLR signaling involved in immune activation in virus-infected bat and human organoids as those shown in Poly(I:C) stimulation experiments (Fig. 3). It required us to fill the gap in the future studies.

Nonetheless, TLR3/RLR inhibitor CYT387 indeed enhanced bat organoid infection of SARS-CoV-2 and CoV-HKU4 more prominently than the human counterparts at the early phase of infections (Fig. 5). Namely, CYT387 enhancement of viral growth adequately reproduced the more rapid and potent induction of antiviral defense demonstrated in bat organoids after Poly(I:C) stimulation. Collectively, the results suggested that, compared to human cells, the higher basal expression of antiviral genes, especially more rapid and potent induction of antiviral response, prepared bat cells to respond to viral infections instantly, which restricted, rather than prevented, viral infections. The more potent host defense gave bat cells an edge to curtail virus propagation in the early phase of infection, which may largely obviate the virus-induced inflammation and immunopathology commonly seen in human coronavirus infections. Ahn et al. demonstrated convincing evidence of a dampened NLRP3-mediated inflammation in bat immune cells in response to RNA viruses.^13^ As such, further investigation of the interplay between bat immune cells and bat intestinal organoids is warranted.

## Materials and methods

### Establishment and maintenance of bat and human intestinal organoids

Horseshoe bats were procured for the derivation of intestinal organoids under ethical approval by the Institutional Review Board of the University of Hong Kong/Hospital Authority Hong Kong West Cluster (CULATR 5431-20) and the Agriculture, Fisheries, and Conservation Department, Government of Hong Kong Special Administrative Region. Bat intestinal organoids were derived as described previously.^24^ In brief, we harvested bat intestines after bats were euthanized by intraperitoneal anesthesia. After washing with cold PBS, intestinal tissues were chopped into small pieces and then digested with 2 mg/ml collagenase (Sigma Aldrich) for 30 mins at 37 °C, followed by shearing using a glass Pasteur pipette (Drummond) and straining over a 100-μm cell strainer (FALCON). The resultant single cells were then pelleted by centrifugation at 200 g for 3 min, resuspended in cold Matrigel (Growth Factor Reduced Basement Membrane Matrix, Corning), and 40 µl of cell suspension was dispersed in a well of a 24-well plate. After Matrigel was polymerized to form a droplet, we dispersed 500 µl culture medium (Supplementary Table 1) to each well and maintained the organoids at 37 °C in a humidified incubator with 5% CO_2_. Bat intestinal organoids were passaged every 7 days with a ratio of 1:3-5, and the culture medium was replenished every other day. Photomicrographs of the organoids were acquired using a Nikon Eclipse TS100 inverted routine microscope. Multiple lines of human intestinal organoids were previously derived from different donors after being approved by the Institutional Review Board of the University of Hong Kong/Hospital Authority Hong Kong West Cluster (UW21-695).^26^

### Construction of stable cell line expressing Wnt surrogate

A plasmid encoding the Wnt surrogate with an Fc tag was kindly provided by Professors Hans Clevers and Christopher Garcia. A stable cell line expressing the Wnt surrogate was established as described previously.^48^ We measured the concentration of Wnt surrogate in the culture media using a human lgG1 Fc ELISA kit (Thermo Fisher Scientific, BMS2092). A stable cell line with the highest expression of human lgG1 Fc was maintained to produce the Wnt surrogate conditioned medium. The activities of Wnt surrogate and Wnt3a conditioned medium were measured in HEK 293 STF cells (ATCC, CRL-3249). The Wnt surrogate conditioned medium was then supplemented in the culture medium for cultivating bat intestinal organoids in a volume with an activity comparable to that of Wnt3a conditioned medium.

### Amplification of full-length cDNA sequences of horseshoe bat IFNL1 and IFNL3

Total RNA was extracted from bat intestinal organoids using an RNeasy kit (Qiagen, 74106) and applied to 5′ and 3′ rapid amplification of cDNA ends (RACE) using the GeneRacer™ Kit (Thermo Fisher Scientific, L150201). To obtain 5′ and 3′ sequences, we amplified first-strand cDNA using gene-specific primers (Supplementary Table 2) and GeneRacer™ 5′ and 3′ primers, respectively. After purification, the PCR amplicons were cloned into the pCR^®^4-TOPO^®^ vector for sequencing. A total of 20 colonies from each amplicon were sequenced. The sequences were aligned and analyzed by ApE DNA software. We have deposited the cDNA sequences of both genes in GenBank with accession numbers OM937883 and OM937884.

### Poly(I:C) stimulation and inhibition experiments

Bat and human organoids were sheared using a glass Pasteur pipette and incubated in a basal medium (Advanced DMEM/F-12 (Gibco) supplemented with 1% HEPES, 1% GlutaMAX and 1% Penicillin/Streptomycin) with 10 µg/ml Poly(I:C) (InvivoGen, tlrl-pic-5) or DMSO in a suspension plate. At the indicated time points post-treatment, treated or mock-treated organoids were harvested and subjected to RT-qPCR assay and Western blot. The cell-free media were collected and applied to ELISA and PRM-MS. For pathway inhibition experiments, bat and human intestinal organoids in the basal medium were pretreated with 1 µg/ml or 0.1 µg/ml CYT 387 (InvivoGen, inh-cy87), or 1 µM or 0.1 µM BX795 (InvivoGen, tlrl-bx7), or mock-treated with 0.1% DMSO overnight. Subsequently, the organoids were sheared mechanically and incubated with or without 10 µg/ml Poly(I:C) and the inhibitors with the initial concentrations in a suspension plate. At the indicated time points, the organoids were collected to detect the expression levels of antiviral genes by RT-qPCR assay. Cell-free media were harvested and applied to ELISA and PRM-MS.

### RT-qPCR assay, western blot, and ELISA

Bat and human intestinal organoids were lysed and subjected to total RNA extraction using the RNeasy kit, followed by reverse transcription using the PrimeScript RT-PCR kit (Takara, PR014B) and an oligo(dT) primer. The resultant cDNAs were used to measure mRNA expression levels of cellular genes using LightCycler 480 SYBR Green I Master Mix (Roche) and an LC480 thermocycler (Roche). Data were analyzed by the delta-delta Ct method. qPCR primers were listed in Supplementary Table 2.

For Western blot, human intestinal organoids were lysed in RIPA buffer supplemented with protease inhibitors (Roche). The lysates were separated in 12% SDS-PAGE and then transferred to a 0.22 µm PVDF membrane (Bio-Rad). After overnight blocking with 5% skimmed milk (Bio-Rad), the membrane was incubated with an anti-ISG15 antibody (Thermo Fisher Scientific, MA5-15029) for 2 h at room temperature, followed by incubation with an HRP-conjugated secondary antibody and detection with immobilon crescendo western HRP substrate (Millipore). Cell-free media were harvested from treated and mock-treated human intestinal organoids for measuring the amount of human IFNL1 and IFNL3 using ELISA kits (R&D Systems, DY7246, D28B00).

### Parallel reaction monitoring-mass spectrometry assay

Cell-free media were harvested from bat and human organoids by acetone precipitation overnight and resuspended in 50 mM TEABC / 8 M urea. Protein samples were then treated with 50 mM TCEP for 30 mins at 55 °C, followed by alkylation with 55 mM IAA for 30 min in the dark. Buffer exchange was performed in a 10 kDa filter unit (Millipore), followed by trypsin digestion for 18 h at 37 °C in 50 mM TEABC (enzyme: protein ratio of 1:100). Sample desalting was performed with a C18 spin-tip (Thermo) before being subjected to MS analysis on a timsTOF Flex Mass Spectrometer (Bruker). A 45-minute gradient (0.2% formic acid in water and 99.8% acetonitrile with 0.2% formic acid) was set according to the manufacturer’s recommendation using a 25 cm × 75 μm × 1.6 μm C18 column (IonOpticks). Default DDA short cycle time settings were used for library construction. The DDA data files were searched against the human UniProt database or in-house bat database using Maxquant (v2.0.3.1). The identified peptides for human ISG15 (LTQTVAHLK), bat ISG15 (IAQETGVPAFQQR) and bat IFNL3 (LLTLDLK) were then chosen to create PRM methods for targeted MS detection in Skyline software (version 21.2). Tryptic digested samples were then analyzed again by the targeted PRM assay using the same nanoLC gradient, which only scanned the targeted m/z precursor with a particular CCS value at a given retention time, at a cycle time of 100 mS. Data were then analyzed by Skyline. Only peptides with Peak Found Ratio of 100% were considered as identified and their total area of MS1 was calculated and exported by Skyline.

### Immunofluorescence staining, flow cytometry, and transmission electron microscopy

Virus-inoculated and mock organoids, after fixation, were applied to immunofluorescence staining using in-house-made antibodies against SARS-CoV-2 NP,^25^ or CoV-HKU4 NP, or EV-71 VP1^26^ and secondary antibodies of goat-anti-rabbit IgG Alexa Fluor 488 (A-11034, Invitrogen) or goat-anti-mouse IgG Alexa Fluor 488 (A-11001, Invitrogen) to label the virus-infected cells. Bat intestinal organoids were also stained with an anti-Villin (Abcam, ab201989) and an anti-MUC2 (Thermo Fisher Scientific, MA5-12345) to identify enterocytes and goblet cells, respectively. Nuclei and actin filaments were counterstained with DAPI (Thermo Fisher Scientific) and Phalloidin-647 (Sigma-Aldrich), respectively. The organoids were whole-mounted on a glass slide with ProLong™ Glass Antifade Mountant (Invitrogen) after staining. Confocal images were acquired using a Carl Zeiss LSM 800 confocal microscope. For flow cytometry analysis, bat and human intestinal organoids were sheared and then incubated with 10 μg/ml Poly(I:C) Fluorescein (InvivoGen) for 2 h. Organoids were collected and digested with 10 µM EDTA (Thermo Fisher Scientific) to make single-cell suspension for flow cytometry to detect the percentage of Fluorescein-positive cells in a BD LSR Fortessa. FlowJo software was used for data processing. For transmission electron microscopy, bat intestinal organoids were embedded in resin after sequential fixation in 2.5% glutaraldehyde and 1% osmium. The ultrathin sections were stained with uranyl acetate and examined under a Philips CM 100 transmission electron microscope.

### Virus infection and detection

A SARS-CoV-2 ancestral strain HKU-001a (WT, GenBank accession number MT230904), and an Omicron variant (B.1.1.529; GenBank OM212473) were propagated in VeroE6/TMPRSS2 cells and titrated with plaque assay. Clinical isolates of EV-71 (GenBank accession number DQ341368.1) were propagated and titrated in RD cells as we described previously.^26^ CoV-HKU4 (GenBank accession number PRJNA251999) was propagated and titrated in Caco-2 cells as described elsewhere.^49^ Bat and human organoids were sheared mechanically and incubated with SARS-CoV-2, or CoV-HKU4, or EV-71 at the indicated MOI at 37 °C for 2 h. After washing, the organoids were re-embedded into Matrigel and then maintained in the basal medium. At the indicated hours after inoculation, cell-free culture media were harvested and applied to RNA extraction using the MiniBEST Viral RNA/DNA Extraction Kit (Takara), detection of viral loads (viral gene copy numbers) by one-step RT-qPCR assay (QuantiNova Probe RT–PCR kit, Qiagen, primers listed in Supplementary Table 2), and viral titration by TCID50 assay, as described previously.^50,51^ After pretreatment with 1 µg/ml CYT387 or mock treatment overnight, bat and human organoids were sheared and inoculated with SARS-CoV-2 or CoV-HKU4 viruses in a suspension plate. After inoculation, the organoids were incubated in the presence or absence of 1 µg/ml CYT387. At the indicated time points post-inoculation, cell-free media were collected and applied to viral load detection and TCID50 assay. To examine cellular response in SARS-CoV-2-infected human and bat organoids, we harvested the organoids at 48 h after a MOI of 2 inoculation or mock inoculation. The organoids were applied to RNA extraction and RT-qPCR assay for detection the induction of related immune genes as described above.

### Data analysis

Statistical analysis was conducted using GraphPad Prism 9.0. Student’s t-test or ANOVA test was used to determine statistical significance as specified in the figure legends. *p ≤ 0.05, **p ≤ 0.01, ***p ≤ 0.001.

## Supplementary information

Analogous comparison unravels heightened antiviral defense and boosted viral infection upon immunosuppression in bat organoids

## Data Availability

All data reported in this paper will be shared by the lead contact upon request. This paper does not report original code. Any other information required to reanalyze the data reported in this paper are available upon request.
