# Supplementary material for: Analogous comparison unravels heightened antiviral defense and boosted viral infection upon immunosuppression in bat organoids
Source: Signal Transduct Target Ther. 2022 Dec 19;7:392. doi: 10.1038/s41392-022-01247-w (PMC9760641; doi:10.1038/s41392-022-01247-w)
Supplement: Supplementary file 1 — Analogous comparison unravels heightened antiviral defense and boosted viral infection upon immunosuppression in bat organoids [file 41392_2022_1247_MOESM1_ESM.docx]

Supplementary Materials for

**Analogous comparison unravels heightened antiviral defense and boosted viral infection upon immunosuppression in bat organoids**

Xiaojuan Liu^1^, Cun Li^1^, Zhixin Wan^1^, Man Chun Chiu^1^, Jingjing Huang^1^, Yifei Yu^1^, Lin Zhu^2^, Jian-Piao Cai^1^ , Lei Rong^3^, You-qiang Song^3,4^, Hin Chu^1,5,6^, Zongwei Cai^2^, Shibo Jiang^7^*, Kwok-yung Yuen^1,5,6^*, Jie Zhou^1,5,6^*

***Correspondence to:** Jie Zhou, Email: [jiezhou@hku.hk](mailto:jiezhou@hku.hk) and Kwok-Yung Yuen, Email: [kyyuen@hku.hk](mailto:kyyuen@hku.hk) and Shibo Jiang, [shibojiang@fudan.edu.cn](file:///D:\1_2022-2-4\3_My%20Documents\1_Meeting\2022-3-21_福庆云学堂讲座\shibojiang@fudan.edu.cn)

**This PDF file includes:**

Supplementary Figures 1-6

Supplementary Tables 1-2


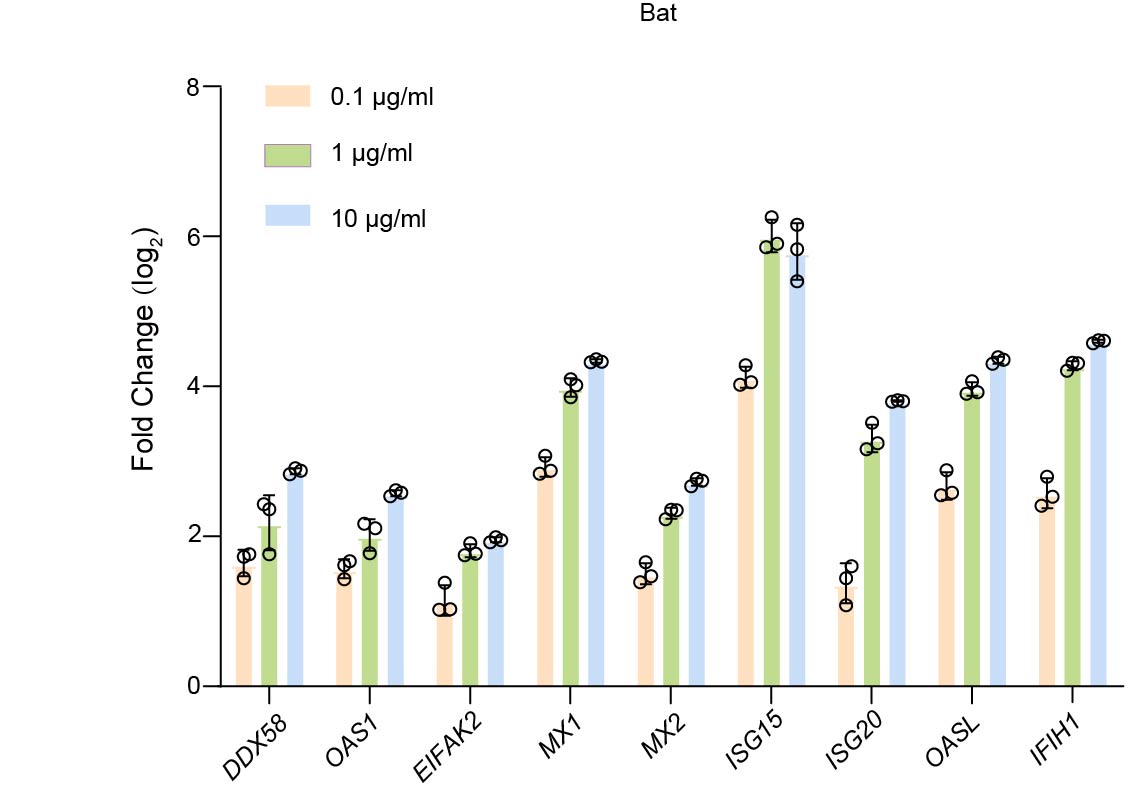


**Supplementary Fig. 1.**

Bat intestinal organoids were mechanically sheared and treated with increasing concentrations of Poly(I:C) (0.1, 1, 10 μg/ml ) or mock-treated. Organoids were collected at 4 hours post-treatment and applied to RT-qPCR to detect bat ISGs. Results show the log2-fold change of GAPDH-normalized expression level in the treated organoids relative to mock-treated organoids. Data represent the mean and s.d. of a representative experiment in organoids derived from a bat donor, n = 3.


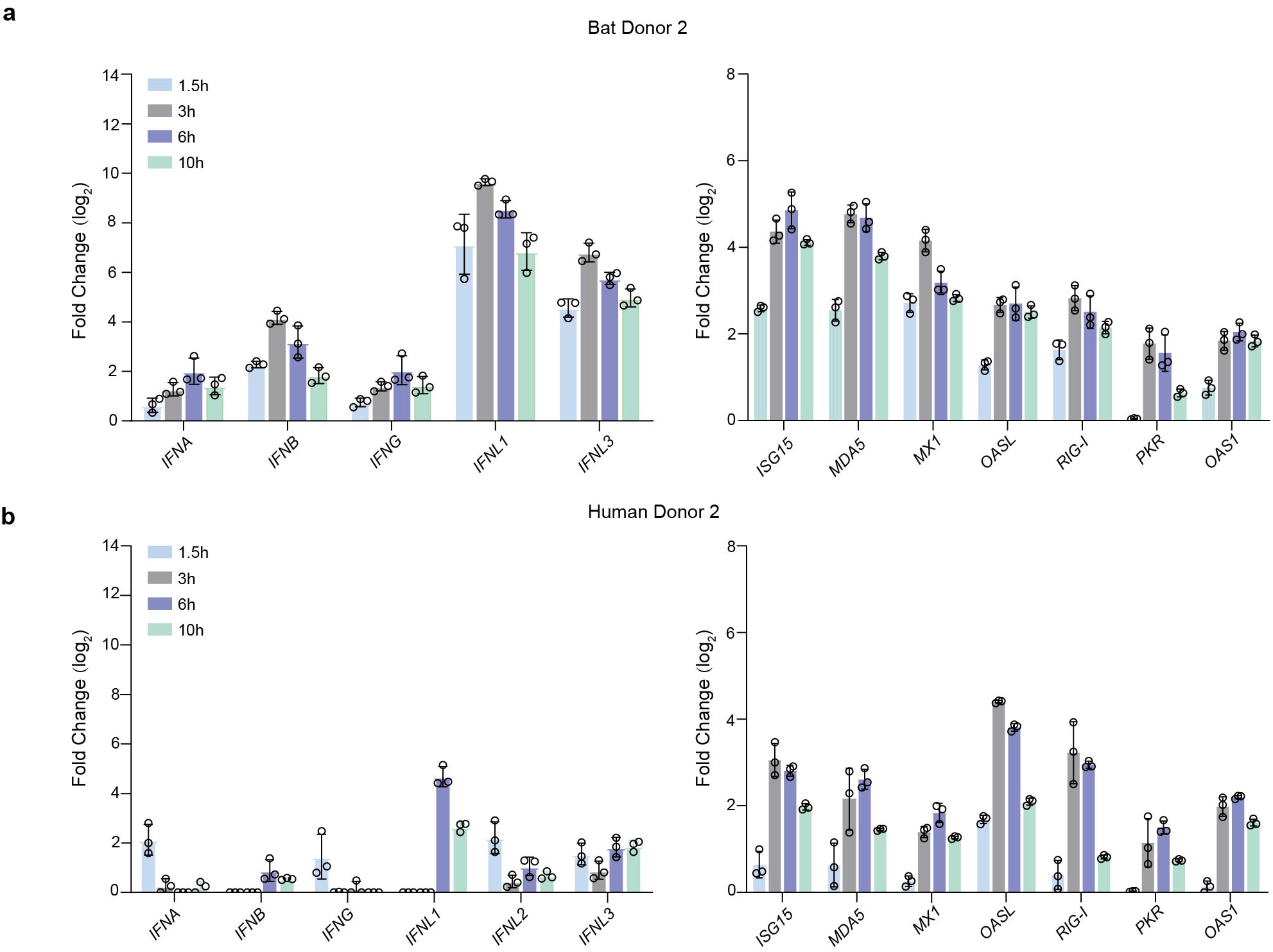


**Supplementary Fig. 2.**

(a, b) Induction of IFNs and ISGs in intestinal organoids derived from another bat donor (a) and another human donor (b) at the indicated time points after Poly(I:C) treatment. Results show the log2-fold change of GAPDH-normalized expression level in the treated organoids relative to mock-treated organoids. Data represent the mean and s.d. of a representative experiment in organoids from a bat and human donor, n = 3.


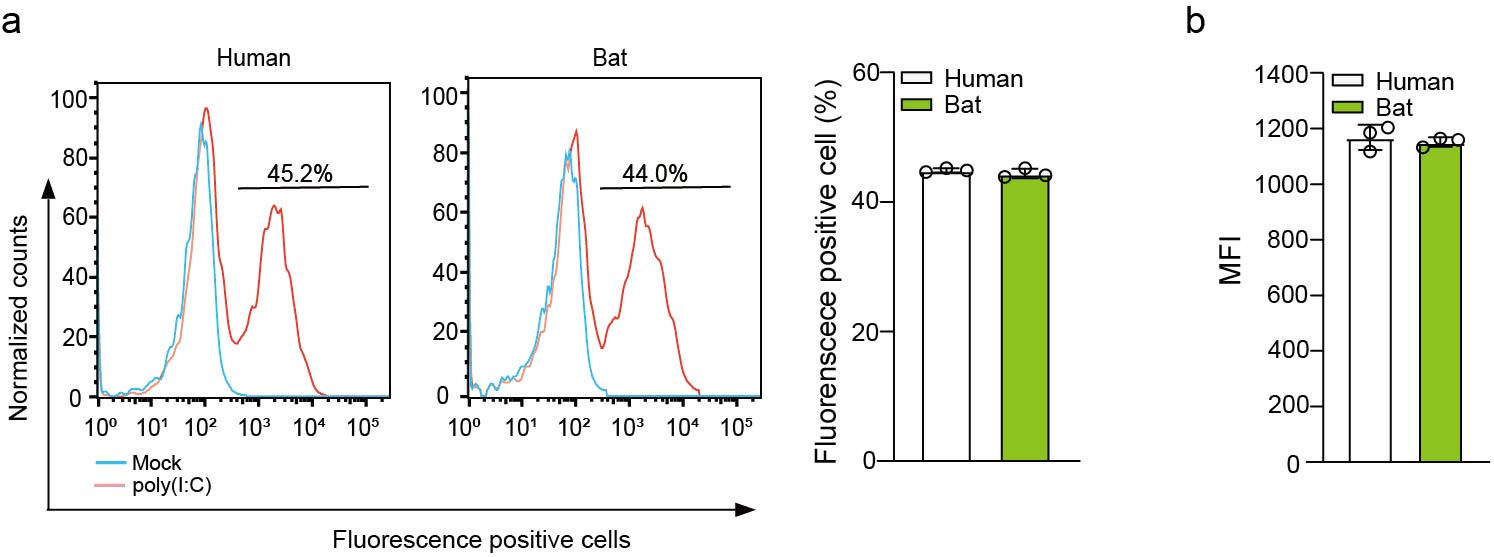


**Supplementary Fig. 3.**

Bat and human intestinal organoids were sheared and incubated with 10 μg/ml Poly(I:C) Fluorescein (InvivoGen) in triplicate for 6 hours. The organoids were then dissociated and applied to flow cytometry to detect the percentage (a) and mean fluorescence intensity (MFI, b) of Fluorescein-positive cells. Data represent the mean and s.d. of a representative experiment in organoids from a bat and human donor, n = 3.


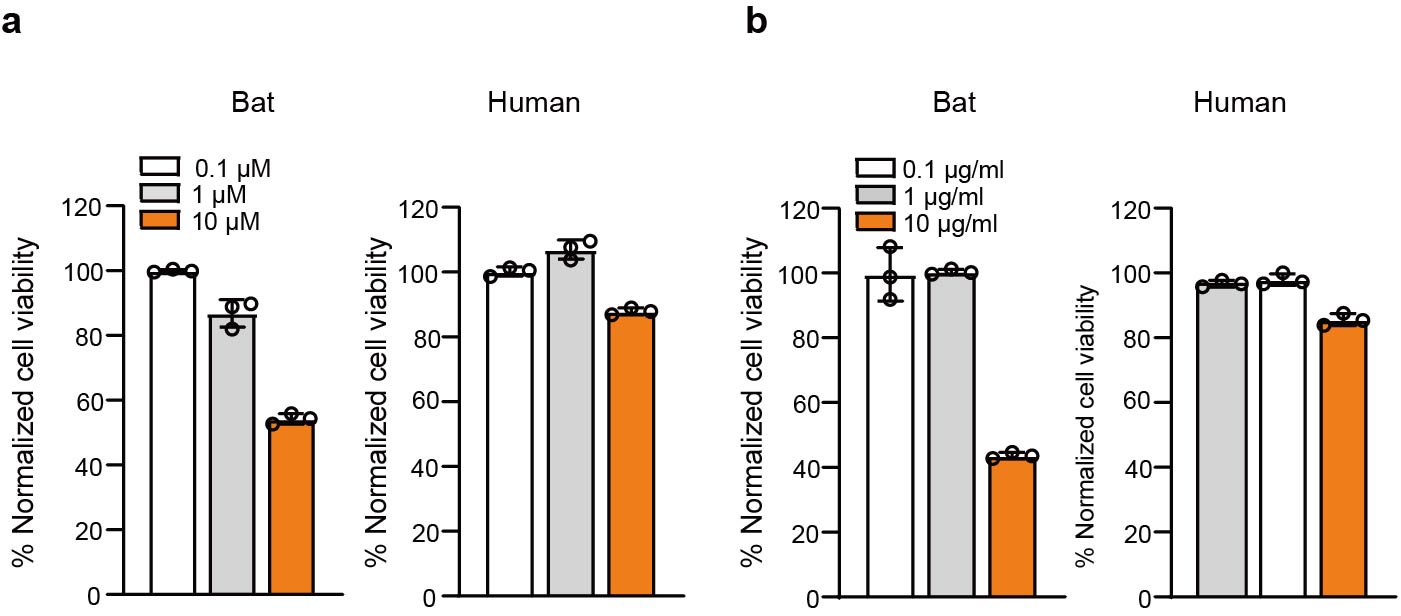


**Supplementary Fig. 4.**

(a, b) Bat and human intestinal organoids were treated with BX795 (a) and CYT387 (b) with the indicated concentrations or mock-treated with DMSO. After overnight incubation, organoids were applied to detect cell viability with CellTiter-Glo Cell viability Assay. Data represent the mean and s.d. of a representative experiment in organoids from a bat and human donor, n = 3.


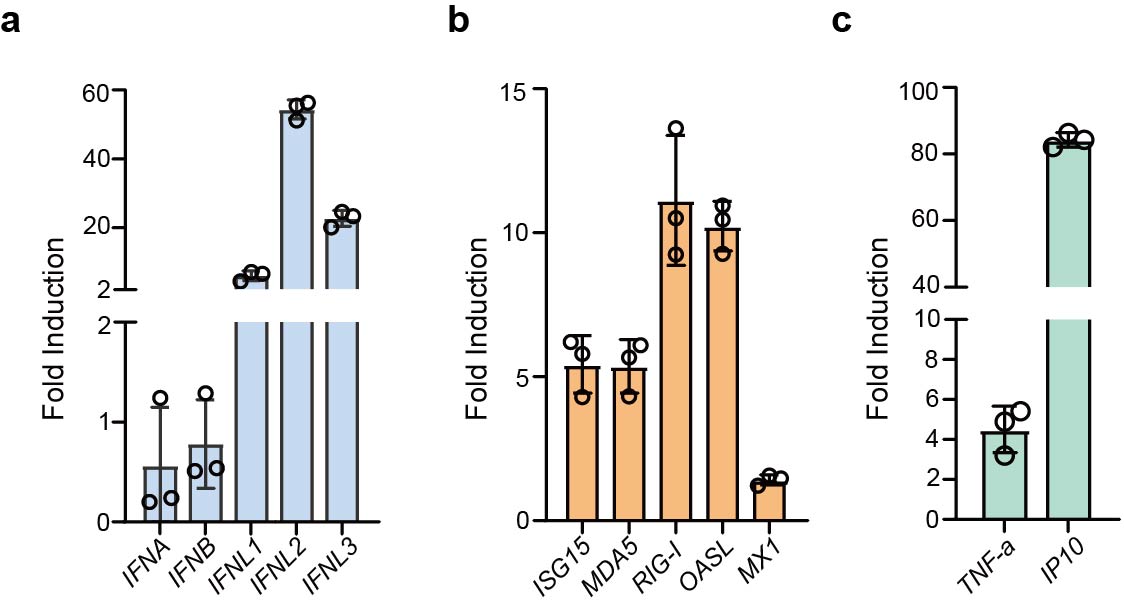


**Supplementary Fig. 5.**

At 48 hours post SARS-CoV-2 inoculation (2 MOI) or mock infection, human intestinal organoids were harvested for examining the mRNA expression of IFNs (a), ISGs(b) and proinflammatory cytokines (c). Results show the fold change of GAPDH-normalized expression level in the infected organoids relative to the mock-infected organoids. Data represent the mean and s.d. of a representative experiment in the organoids from one donor, n = 3.


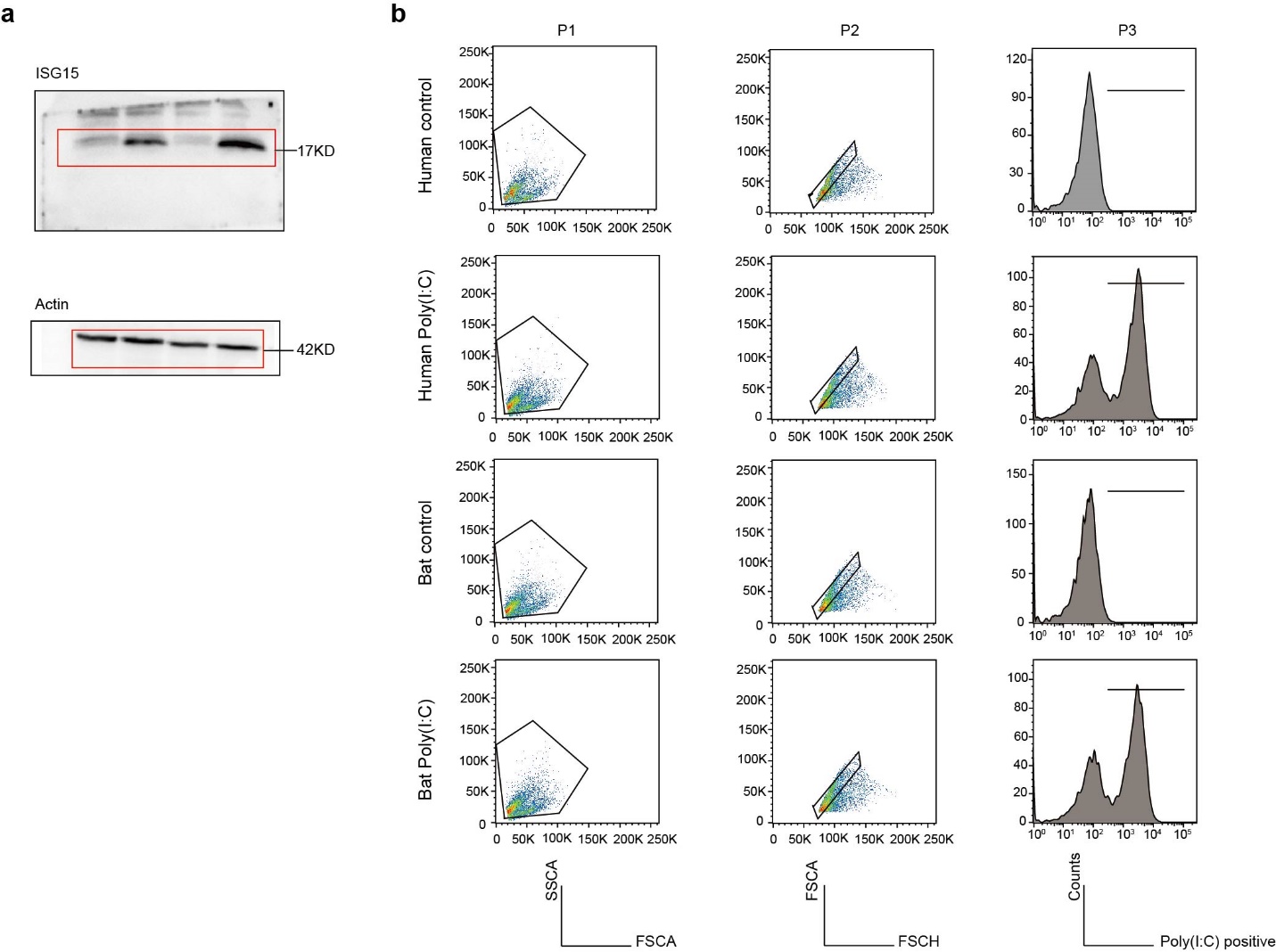


**Supplementary Fig. 6.**

(a) Original and uncropped films of Western blots in Fig. 2d. (b) Gating strategies of the flow cytometry data in Fig. 2e-f.

**Supplementary Table 1. Composition of the organoid culture medium**

| Reagents | Company | Catalog No. | Working concentration |
| --- | --- | --- | --- |
| Advanced DMEM/F12 | Gibco | 12634010 | - |
| HEPES | Gibco | 15630056 | 1% |
| GlutaMAX | Gibco | 35050061 | 1% |
| P/S | Gibco | 15140122 | 1% |
| Rspondin1 (conditioned medium) | - | - | 20% |
| Noggin (conditioned medium) | - | - | 10% |
| Wnt surrogate (conditioned medium) |  |  | 5% |
| B27 supplement | Gibco | 17504044 | - |
| N-acetylcysteine | Sigma-Aldrich | A9165 | 1.25 mM |
| Nicotinamide | Sigma-Aldrich | N0636 | 10 mM |
| Y-27632 | Tocris | 1254 | 5 µM |
| A8301 | Tocris | 2939 | 500 nM |
| SB202190 | Sigma-Aldrich | S7067 | 1 µM |
| hEGF | Peprotech | AF-100-15 | 50 ng/ml |
| hGastrin I | Sigma-Aldrich | G9145 | 10 nM |
| Primocin | InvivoGen | ant-pm-1 | 100 µg/ml |

**Supplementary Table 2.**

| Gene | Sequences | | | | | |
| --- | --- | --- | --- | --- | --- | --- |
| Bat *IFNL1* 5’ GSP | | CATCTCCTCGGCCTTATGAATCCTG | | | | |
| Bat *IFNL1* 3' GSP | | CAAGACTCTGTCTCCACAAGAGC | | | | |
| Bat IFNL3 5' GSP | | CGACTCTTCACACAGGTCTCCAC | | | | |
| Bat *IFNL3* 3' GSP | | TTCAAGTCTCTGTCTCCACAAGAGC | | | | |
| Bat *ISG15* | | F: | AAGAACGAGAAGGGTCGCAC; | | R: | AAACTCAGCCAGAACTGGTC |
| Bat *TNF-a* | | F: | ATCTACTCCCAGGTCCTCTTC; | | R: | AAGGGCTCTTGATGGCAGAC |
| Bat *IP10* | | F: | TCACGAGGAGTGATAAACCTG; | | R: | GGTGGTCTTAGACTCTGGATTC |
| Bat *IL6* | | F: | CCTGACCCAATCACAAGCTCC; | | R: | GAACAGCTCTCTGGGCGAAC |
| Bat *OASL* | | F: | CTGTATGGCACCTCTGCCTC; | | R: | TCTCTTCCCTCAGAAACTGCTCC |
| Bat *RIG-I* | | F: | TACAAGGAAGAACCAGAATGCCAG; | | R: | GTGTTTCTTCCTTGCTTAGCAG |
| Bat *MDA5* | | F: | GCATTCCTCAGACAAGAGTTTCC; | | R: | CTCAATCTTCTCCTTCTCATCTGC |
| Bat *OAS1* | | F: | TTCATCCAGGACCACCTGCTAC; | | R: | CCTTCACGACTTTGGACACC |
| Bat *MX1* | | F: | ATCCTTAGGCAGGAGACGATCAAC; | | R: | GTCAGGCTTCGTCAGGATTC |
| Bat *IFNG* | | F: | GCCAGGTTGTTTCCTTCTACTTC; | | R: | TTCCAATTTACTGCTGCTGC |
| Bat *GAPDH* | | F: | CGTATTGGACGCCTGGTCAC; | | R: | TGGGTGGAATCATACTGGAAC |
| Bat *IFNA* | | F: | TGCATCTGAACCATGTCCTG; | | R: | CTGCTATCTACTGTCTCCTGG |
| Bat *IFNB* | | F: | TGACCAGCCAGTCCATCCTTC; | | R: | GCCACAGGAGGTTCTGACAAG |
| Bat *IFNL1* | | F: | TTTCCACCTCAACCAGGACG; | | R: | CTGAAGCAGCAGCTCTGAGG |
| Bat *IFNL3* | | F: | TGGCTACACAGGATTCATAAGG; | | R: | TCTCCACTGGCAACACATTTC |
| Bat *EIFAK2* | | F: | CCAGAAGCTGAAGGTAGATCAAAG; | | R: | TTCCCAGCGGTTAAATCTGAAG |
| Human *IFNA* | | F: | CTTGAAGGACAGACATGACTTTGGA; | | R: | GGATGGTTTCAGCCTTTTGGA |
| Human *IFNB* | | F: | CAACTTGCTTGGATTCCTACAAAG; | | R: | TATTCAAGCCTCCCATTCAATTG |
| Human *IFNG* | | F: | CTAATTATTCGGTAACTGACTTGA; | | R: | ACAGTTCAGCCATCACTTGGA |
| Human *IFNL1* | | F: | CACATTGGCAGGTTCAAATCTCT; | | R: | CCAGCGGACTCCTTTTTGG |
| Human *IFNL2* | | F: | TCCAGTCACGGTCAGCA; | | R: | CAGCCTCAGAGTGTTTCTTCT |
| Human *IFNL3* | | F: | TAAGAGGGCCAAAGATGCCTT; | | R: | CTGGTCCAAGACATCCCCC |
| Human *TNF-a* | | F: | GGCTCCAGGCGGTGCTTGTTC; | | R: | AGACGGCGATGCGGCTGATG |
| Human *IP10* | | F: | GAAATTATTCCTGCAAGCCAATTT; | | R: | TCACCCTTCTTTTTCATTGTAGCA |
| Human *IL6* | | F: | GGTACATCCTCGACGGCATCT; | | R: | GTGCCTCTTTGCTGCTTTCAC |
| Human *MX1* | | F: | GCCAGGACCAGGTATACAG; | | R: | GCTCCTTCAGGAGCCAGA |
| Human *OAS1* | | F: | CATCCGCCTAGTCAAGCACTG; | | R: | CACCACCCAAGTTTCCTGTAG |
| Human *OASL* | | F: | GTACCAGCAGTATGTGAAAG; | | R: | ATGGTTAGAAGTTCAAGAGC |
| Human *RIG-I* | | F: | AGGAAAACTGGCCCAAAACT; | | R: | TTTCCCCTTTTGTCCTTGTG |
| Human *MDA5* | | F: | GTGCATGGAGGAGGAACTGT; | | R: | GTTATTCTCCATGCCCCAGA |
| Human *ISG15* | | F: | GAGAGGCAGCGAACTCATCT; | | R: | AGGGACACCTGGAATTCGTT |
| Human *GAPDH* | | F: | GGAGCGAGATCCCTCCAAAAT; | | R: | GGCTGTTGTCATACTTCTCATGG |
| SARS-CoV-2 | | F: | CGCATACAGTCTTRCAGGCT; | | R: | GTGTGATGTTGAWATGACATGGTC |
|  |  | Probe: FAM-TTAAGATGTGGTGCTTGCATACGTAGAC-lABkFQ | | | | |
| CoV-HKU4 | | F: | | CGGAAAATCAACACCGGTAATGGT; | R: | TAGCC TCTGGTCCAGTCCCA |
|  |  | Probe: FAM-TTAAACAATTGGCYCCCAGATGGTTCTTCTA CTACA-BHQ1 | | | | |
| EV-71 | | F: | | GCCCCTGAATGCGGCTAAT; | R: | ATTGTCACCATAAGCAGYCA |
|  |  | Probe: FAM-CGGACACCCAAAGTAGTCGGTTCCG-lABkFQ | | | | |
